# Analysis of depressive symptom trajectory patterns with zuranolone: Time‐series clustering of Japanese Phase 2 and Phase 3 trial data

**DOI:** 10.1002/pcn5.70320

**Published:** 2026-03-13

**Authors:** Masaki Kato, Takamichi Baba, Saki Nakano, Yuto Kashiwagi, Tomoko Motomiya, Nakao Iwata

**Affiliations:** ^1^ Department of Neuropsychiatry Kansai Medical University Osaka Japan; ^2^ Drug Development and Regulatory Science Division Shionogi & Co., Ltd. Osaka Japan; ^3^ Data Science Department Shionogi & Co., Ltd. Osaka Japan; ^4^ Medical Affairs Department Shionogi & Co., Ltd. Osaka Japan; ^5^ Department of Psychiatry Fujita Health University School of Medicine Aichi Japan

**Keywords:** Japan, major depressive disorder, post hoc analysis, symptom trajectory, zuranolone

## Abstract

**Aim:**

Major depressive disorder (MDD) shows varied treatment responses. Previous Phase 2 and 3 trials demonstrated the efficacy of 2‐week oral zuranolone in Japanese MDD patients, but individual symptom trajectories were not analyzed. To address this gap, this study aimed to characterize patterns of depressive symptom trajectories among these patients.

**Methods:**

We conducted a pooled analysis of Phase 2/3 randomized, double‐blind, placebo‐controlled trials in Japanese adults with MDD. Participants received zuranolone 30 mg or placebo once daily for 14 days, with a 6‐week follow‐up. Patients with complete 17‐item Hamilton Rating Scale for Depression (HAM‐D17) data at baseline and 10 subsequent time points through Day 57 were included. Change from baseline scores underwent Dynamic Time Warping–based k‐means clustering identified symptom trajectory patterns in the zuranolone group, with optimal cluster number determined by the elbow method. A Random Forest classifier then assessed scale items linked to symptom worsening trajectories.

**Results:**

Four distinct trajectories were identified. Three clusters showed varying degrees of symptom reduction during treatment, stabilizing during follow‐up. The fourth cluster showed symptom reduction during the treatment period and a mean increase in HAM‐D17 scores after Day 15, primarily due to worsening insomnia. Each Patient Health Questionnaire‐9 (PHQ‐9) score showed a similar course to the corresponding HAM‐D17 scores. No notable differences were observed among clusters in baseline demographics, episode duration, or initial severity.

**Conclusion:**

This study reveals diverse symptom trajectories in zuranolone‐treated patients and provides clinically relevant insights, highlighting the importance of early post‐dosing monitoring—particularly of sleep symptoms—to inform clinical management and future research.

## Graphical abstract

In this pooled analysis of Phase 2/3 trials in Japanese adults with major depressive disorder (MDD), we aimed to characterize depressive symptom trajectories following zuranolone 30 mg administration. Dynamic Time Warping–based k‐means clustering identified four distinct symptom trajectories. While three clusters showed varying degrees of symptom reduction that stabilized during follow‐up, the fourth cluster experienced a decrease in symptom scores during the 14‐day dosing period followed by a mean increase in 17‐item Hamilton Rating Scale for Depression (HAM‐D17) scores after Day 15, primarily driven by worsening insomnia.

## INTRODUCTION

Major depressive disorder (MDD) significantly reduces the quality of life of patients and requires timely intervention to prevent functional decline.^1^ In Japan, the 12‐month prevalence of MDD is 2.7%, and the lifetime prevalence is 5.7%, highlighting its impact on public health.^2^ Clinical practice demands rapid symptom relief to restore daily functioning; however, conventional antidepressants often take several weeks to months to produce a therapeutic effect. During this delay, patients remain vulnerable to mood disturbances, sleep disruption, and impaired cognition. The gap between treatment initiation and observable improvement underscores the unmet need for therapies that accelerate symptom reduction.^3^

Zuranolone is a novel oral antidepressant that acts as a positive modulator of the gamma‐aminobutyric acid (GABA)‐A receptor. It has been approved in the United States and Europe for the treatment of postpartum depression (PPD) in adults.^4^, ^5^ In Japan, two randomized, double‐blind, placebo‐controlled trials in Japanese adults with MDD evaluated the efficacy and safety of 30 mg of zuranolone administered over 14 days, followed by a 6‐week follow‐up period.^6^, ^7^ The Phase 2 trial revealed that treatment with zuranolone demonstrated a significant reduction in the 17‐item Hamilton Rating Scale for Depression (HAM‐D17) total score on Day 15 compared with placebo, with a tolerable safety profile.^5^ The Phase 3 trial corroborated these findings, demonstrating early symptom improvement during the treatment phase and no worsening in mean symptom scores during the follow‐up period.^7^ Based on these results, the Japanese Ministry of Health, Labour, and Welfare approved zuranolone for MDD in December 2025.^8^

While zuranolone has demonstrated efficacy in clinical trials, these findings are based on average changes in outcome measures, which obscure how individual patients' symptoms evolve over time. This lack of granularity represents an important gap, particularly given zuranolone's distinctive dosing schedule—2 weeks of treatment followed by at least a 6‐week drug‐free interval. Understanding individual symptom trajectories is essential before discussing shared decision making (SDM), which plays a critical role in determining whether to continue treatment with zuranolone and how to manage the drug‐free phase. To address this gap, the present post hoc analysis describes patterns of change in depressive symptoms among patients who received zuranolone during the clinical trial period. Specifically, we aimed to classify symptom trajectory patterns across the entire 8‐week study period, encompassing both 2‐week treatment and 6‐week follow‐up phases, and examine their relationship with characteristic symptoms and patient backgrounds. These insights are intended to support treatment planning and patient communication in real‐world clinical practices.

## METHODS

### Study design and data source

This investigation represents a post hoc pooled analysis of two randomized, double‐blind, placebo‐controlled trials conducted in Japan to assess the efficacy and safety of zuranolone monotherapy in adults with MDD. The Phase 2 trial (jRCT2080225180) randomized participants in a 1:1:1 ratio to receive oral zuranolone 20 mg, zuranolone 30 mg, or placebo once daily for 14 days.^6^ Following the double‐blind treatment period, a 6‐week follow‐up without the study drug evaluated the durability of the response. The primary endpoint was the change from baseline in the HAM‐D17 total score on Day 15. The Phase 3 trial (jRCT2031210577) comprised two sequential parts^7^: Part A replicated the randomized, double‐blind, placebo‐controlled design, assigning participants in a 1:1 ratio to zuranolone 30 mg or placebo for 14 days, followed by the same 6‐week follow‐up. Part B served as an open‐label extension for participants who completed Part A, permitting repeat dosing as clinically indicated.

### Participants

The majority of the eligibility criteria for the Phase 2 and Phase 3 trials are the same. Eligible participants in both trials were Japanese adults aged 18–75 years who met the DSM‐5 (Diagnostic and Statistical Manual of Mental Disorders, Fifth Edition) criteria for MDD, with a current depressive episode lasting at least 8 weeks but not more than 12 months before signing the informed consent form. Entry criteria required a baseline HAM‐D17 total score of 22 or higher and a Patient Health Questionnaire‐9 (PHQ‐9) total score of at least 15 on Day 1. The exclusion criteria included serious comorbid medical conditions, treatment‐resistant depression, use of concomitant antidepressants within 14 days prior to Day 1, and active suicidal ideation. For the present analysis, we included participants from the full analysis set (FAS) who were randomized to the zuranolone 30 mg or placebo arms in the Phase 2 and Phase 3 Part A trials and who had complete HAM‐D17 assessments at all required time points: baseline, Days 3, 8, 15, 22, 29, 36, 43, 50, and 57.

### Statistical analysis

In the zuranolone 30 mg arm, we calculated the change from baseline in the HAM‐D17 total score at each time point and applied time‐series clustering to explore distinctive trajectories. We employed Dynamic Time Warping (DTW)–based Time Series K‐means clustering, as implemented in the tslearn (version 0.6.3) Python library.^9^ The DTW distance accommodates nonlinear temporal shifts between two time series, allowing the alignment of similar response patterns occurring at different times. We adopted this approach to account for potential individual differences in treatment response and the timing of symptom changes. DTW‐based Time Series K‐means has been widely used for time‐series classification in medical datasets.^10^, ^11^ To determine the optimal number of clusters, we used the elbow method on the within‐cluster sum of squares for each number of clusters and selected the number of clusters at which further increases yielded minimal improvement in the within‐cluster sum of squares. We also used the silhouette method on the mean silhouette scores for each number of clusters to support the cluster number determination. An identical clustering procedure was used as a supplementary analysis for the placebo arm.

To describe each trajectory cluster, we examined demographic and clinical baseline characteristics, longitudinal changes in efficacy measures—including HAM‐D17 assessed by investigator and PHQ‐9 total scores assessed by patient—and individual symptom item trajectories for both HAM‐D17 and PHQ‐9. We then used a Random Forest classifier to identify the symptom items that contributed most to cluster differentiation. We implemented RandomForestClassifier in scikit‐learn (version 1.5.2) with variable importance scores quantified each symptom's contribution to cluster separation. All descriptive statistics were conducted using SAS version 9.4 (SAS Institute Inc.), and clustering and machine learning analyses were performed using Python version 3.12.6.

## RESULTS

### Patient disposition

**Figure 1:**
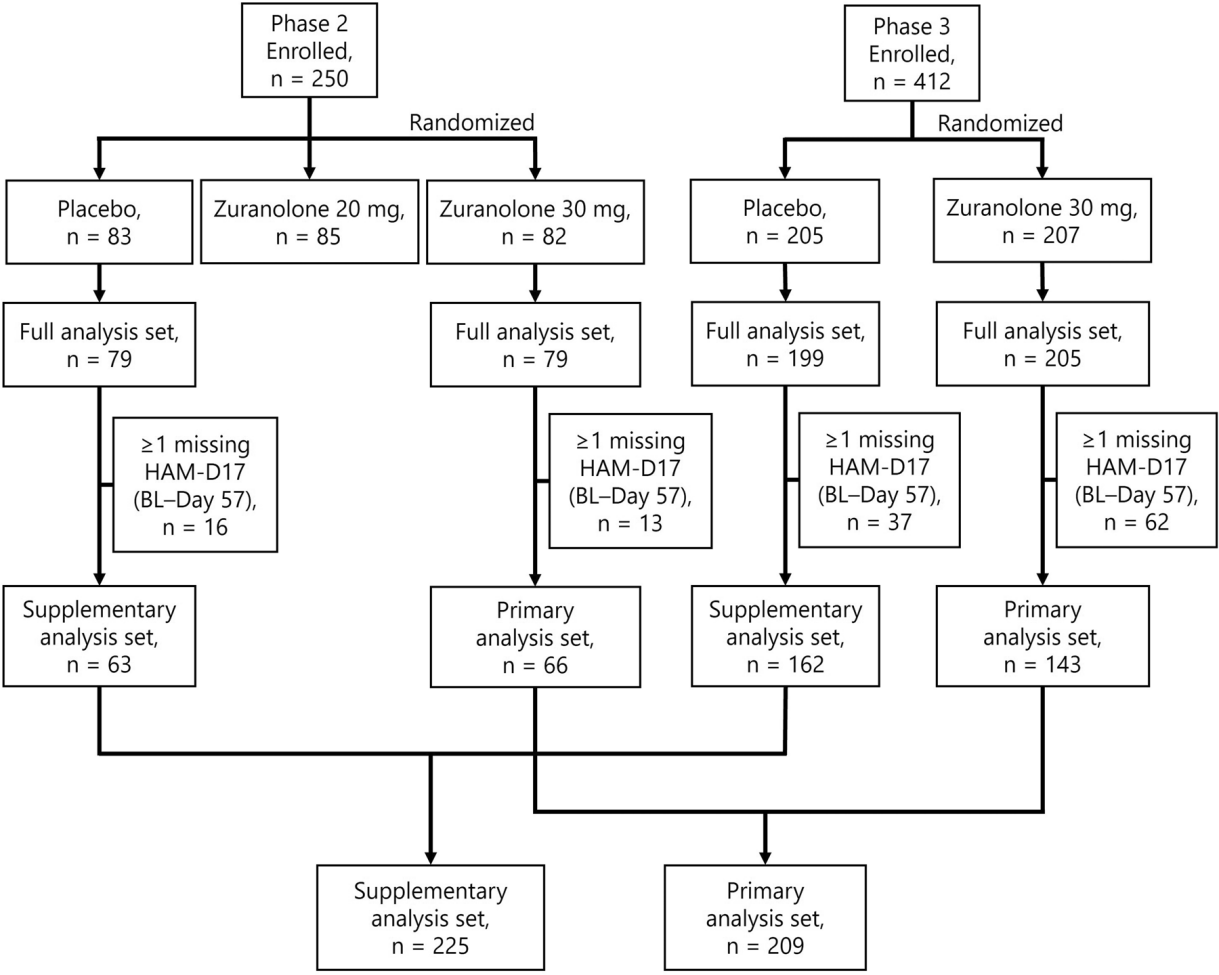
Patient disposition and flow through Phase 2 and Phase 3 trials. BL, baseline; HAM‐D17, 17‐item Hamilton Rating Scale for Depression.

In the Phase 2 trial, 250 patients were randomized to receive placebo (*n* = 83), zuranolone 20 mg (*n* = 85), or zuranolone 30 mg (*n* = 82). Sixty‐six patients in the zuranolone 30 mg group and 63 patients in the placebo group had complete HAM‐D17 assessments at all scheduled visits and were included in the primary and supplementary analysis sets, respectively. In the Phase 3 trial, 412 patients were randomized to receive a placebo (*n* = 205) or zuranolone 30 mg (*n* = 207). Of these, 162 placebo patients and 143 zuranolone 30 mg patients had complete HAM‐D17 data points. Across both trials, the primary analysis set included 209 patients who received zuranolone 30 mg, and the supplementary set comprised 225 patients who received a placebo. Details of patient flow in the Phase 2 and Phase 3 trials are provided in Figure 1. To assess selection bias resulting from the exclusion of 75 patients due to missing HAM‐D17 total scores, we compared demographics and baseline clinical characteristics of the primary analysis set (*n* = 209) and the patients excluded from the primary analysis set (*n* = 75) descriptively. It was confirmed there was no clinically relevant difference between these two groups (Tables S1 and S2).

### Time‐series clustering of HAM‐D17 trajectories

**Figure 2:**
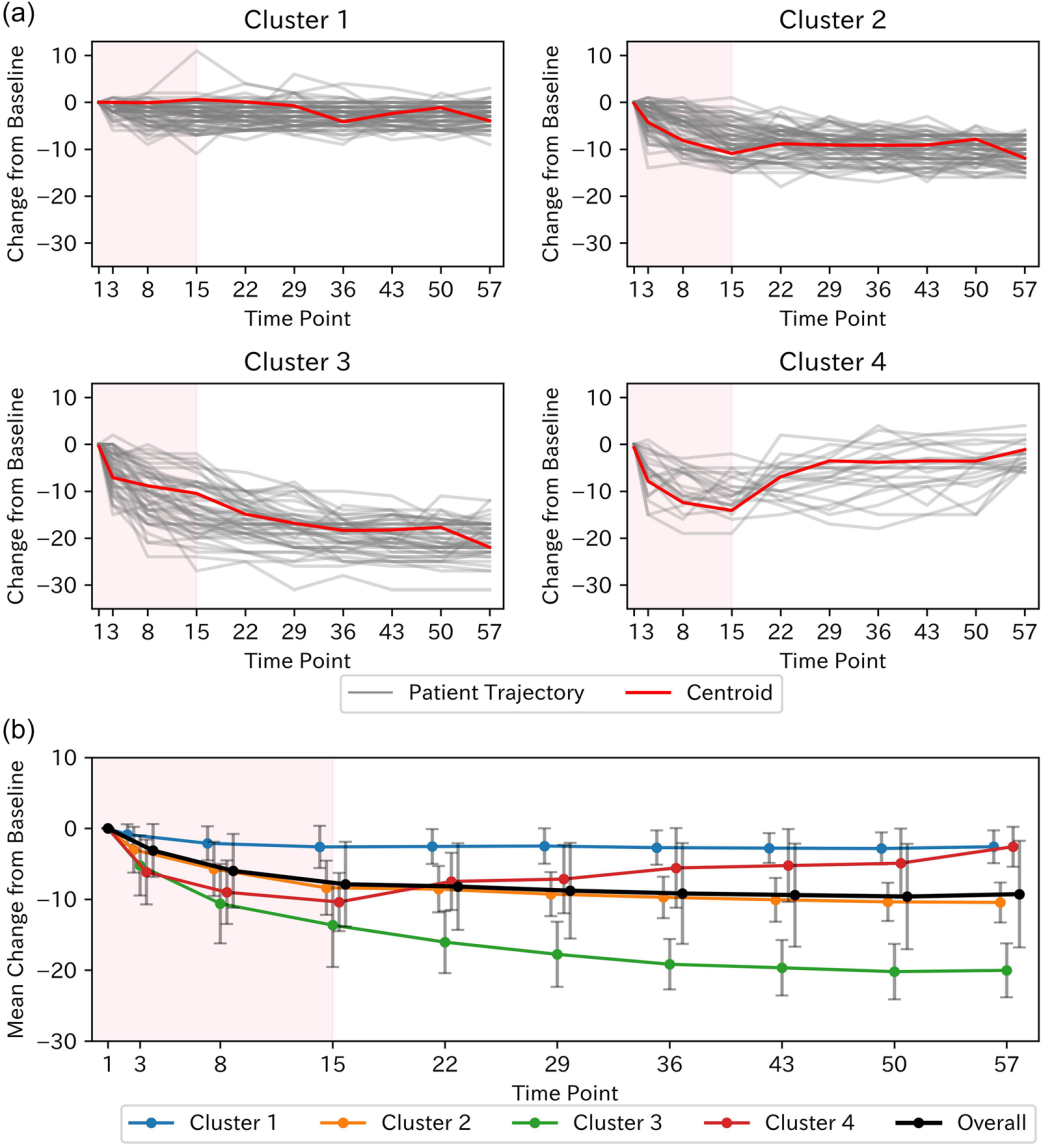
Clustering of 17‐item Hamilton Rating Scale for Depression (HAM‐D17) change from baseline trajectories in the zuranolone 30 mg arm. (a) Individual trajectories and cluster centroids. (b) Mean change from baseline by cluster. Cluster 1 (*n* = 71), Cluster 2 (*n* = 67), Cluster 3 (*n* = 50), and Cluster 4 (*n* = 21). The shaded red area indicates the treatment period (Days 1–15). Error bars in (b) represent standard deviation.

We applied DTW‐based k‐means clustering to change from baseline HAM‐D17 scores in the zuranolone 30 mg arm. The elbow method indicated an optimal cluster count of four clusters. The silhouette method indicated high mean silhouette scores for 2–4 clusters, supporting four clusters as optimal (Figure S1). Both the elbow and silhouette methods consistently identified this optimal cluster count across different initial centroid values. The individual trajectories and centroids by cluster and the mean change profiles are summarized in Figure 2a,b. Cluster 1 (*n* = 71), Cluster 2 (*n* = 67), and Cluster 3 (*n* = 50) showed a larger magnitude of symptom reduction during the 14‐day dosing period in this order; the trajectory of Cluster 1 showed little variation throughout the entire time, and Cluster 2 and Cluster 3 reached a plateau from Day 15 to Day 57 on average. Cluster 4 (*n* = 21) showed a decrease in symptom scores during the 14‐day dosing period similar to Cluster 2 and Cluster 3 and exhibited a mean increase in the HAM‐D17 scores between Days 15 and 57. In the placebo arm, elbow analysis yielded three clusters. The silhouette method indicated high mean silhouette scores for 2–5 clusters, supporting three clusters as optimal (Figure S2). Both the elbow and silhouette methods consistently identified this optimal cluster count across different initial centroid values. None displayed a pattern analogous to Cluster 4 in the zuranolone group (Figure S3).

### Baseline characteristics by cluster

The demographic and clinical features by trajectory cluster for the zuranolone 30 mg arm are shown in Tables 1 and 2. There were no meaningful differences in demographics or baseline disease characteristics across the four clusters.

**Table 1:** Baseline demographic and clinical characteristics by cluster.

		Cluster 1 *N* = 71 *n* (%)	Cluster 2 *N* = 67 *n* (%)	Cluster 3 *N* = 50 *n* (%)	Cluster 4 *N* = 21 *n* (%)	Total *N* = 209 *n* (%)
Sex	Male	32 (45.1)	34 (50.7)	24 (48.0)	9 (42.9)	99 (47.4)
	Female	39 (54.9)	33 (49.3)	26 (52.0)	12 (57.1)	110 (52.6)
Age	Mean (SD)	39.5 (12.1)	40.0 (11.4)	36.9 (10.6)	40.5 (13.3)	39.1 (11.7)
	≥18 to <25	8 (11.3)	8 (11.9)	8 (16.0)	3 (14.3)	27 (12.9)
	≥25 to <45	39 (54.9)	36 (53.7)	30 (60.0)	10 (47.6)	115 (55.0)
	≥45 to <65	23 (32.4)	23 (34.3)	12 (24.0)	8 (38.1)	66 (31.6)
	≥65	1 (1.4)	0	0	0	1 (0.5)
BMI	Mean (SD)	23.54 (5.60)	23.21 (4.01)	21.95 (3.61)	24.17 (6.26)	23.12 (4.80)
Classification based on DSM‐5	Single episode	30 (42.3)	29 (43.3)	21 (42.0)	5 (23.8)	85 (40.7)
	Recurrent	41 (57.7)	38 (56.7)	29 (58.0)	16 (76.2)	124 (59.3)
Episode recurrences	First time	30 (42.3)	30 (44.8)	21 (42.0)	5 (23.8)	86 (41.1)
	Second time	22 (31.0)	16 (23.9)	19 (38.0)	9 (42.9)	66 (31.6)
	Third to seventh time	18 (25.4)	18 (26.9)	10 (20.0)	7 (33.3)	53 (25.4)
	No less than 8 times	0	2 (3.0)	0	0	2 (1.0)
	Unknown	1 (1.4)	1 (1.5)	0	0	2 (1.0)
Duration of current episode at randomization (month)	Mean (SD)	6.7 (2.9)	5.8 (2.7)	6.1 (3.4)	5.8 (2.9)	6.2 (3.0)
	2–4 months	18 (25.4)	22 (32.8)	19 (38.0)	8 (38.1)	67 (32.1)
	4–6 months	15 (21.1)	21 (31.3)	10 (20.0)	4 (19.0)	50 (23.9)
	6–8 months	17 (23.9)	9 (13.4)	3 (6.0)	6 (28.6)	35 (16.7)
	8–10 months	8 (11.3)	6 (9.0)	9 (18.0)	0	23 (11.0)
	10–12 months	10 (14.1)	9 (13.4)	6 (12.0)	2 (9.5)	27 (12.9)
	12 months or more	3 (4.2)	0	3 (6.0)	1 (4.8)	7 (3.3)
Presence or absence of prior drug for depressive episodes	Yes	35 (49.3)	35 (52.2)	30 (60.0)	14 (66.7)	114 (54.5)
	No	36 (50.7)	32 (47.8)	20 (40.0)	7 (33.3)	95 (45.5)

*Note*: Duration of the current episode at randomization = (date of randomization) – (onset date) + 1.

Abbreviations: BMI, body mass index; DSM‐5, Diagnostic and Statistical Manual of Mental Disorders, fifth edition; SD, standard deviation.

**Table 2:** Baseline clinical characteristics by cluster.

Baseline values of HAM‐D17		Cluster 1 *N* = 71 *n* (%)	Cluster 2 *N* = 67 *n* (%)	Cluster 3 *N* = 50 *n* (%)	Cluster 4 *N* = 21 *n* (%)	Total *N* = 209 *n* (%)
Total score	Mean (SD)	23.9 (1.6)	23.9 (1.9)	25.0 (2.4)	24.7 (2.1)	24.2 (2.0)
Depressed mood	Mean (SD)	3.0 (0.6)	2.9 (0.6)	2.8 (0.7)	2.8 (0.7)	2.9 (0.6)
Feelings of guilt	Mean (SD)	1.6 (0.8)	1.4 (0.7)	1.7 (0.7)	1.8 (1.0)	1.6 (0.8)
Suicide	Mean (SD)	0.7 (0.7)	0.7 (0.6)	0.8 (0.7)	0.8 (0.7)	0.7 (0.7)
Insomnia early—early night	Mean (SD)	1.7 (0.7)	1.6 (0.7)	1.7 (0.6)	1.7 (0.7)	1.6 (0.6)
Insomnia middle—middle night	Mean (SD)	1.4 (0.6)	1.4 (0.6)	1.5 (0.5)	1.5 (0.5)	1.4 (0.6)
Insomnia early hours—morning	Mean (SD)	1.2 (0.7)	1.3 (0.7)	1.3 (0.6)	1.6 (0.7)	1.3 (0.7)
Work and activities	Mean (SD)	2.8 (0.6)	3.0 (0.7)	2.9 (0.7)	2.9 (0.8)	2.9 (0.7)
Retardation	Mean (SD)	1.1 (0.8)	1.1 (0.7)	1.2 (0.7)	1.3 (0.6)	1.2 (0.7)
Agitation	Mean (SD)	0.8 (0.7)	0.9 (0.7)	0.9 (0.6)	0.9 (0.7)	0.9 (0.7)
Anxiety psychic	Mean (SD)	2.4 (0.9)	2.3 (0.8)	2.5 (0.7)	2.4 (0.8)	2.4 (0.8)
Anxiety somatic	Mean (SD)	1.6 (0.6)	1.8 (0.7)	1.8 (0.7)	1.9 (0.6)	1.7 (0.7)
Somatic symptoms gastrointestinal	Mean (SD)	0.9 (0.5)	0.9 (0.6)	0.9 (0.7)	0.8 (0.6)	0.9 (0.6)
General somatic symptoms	Mean (SD)	1.5 (0.5)	1.5 (0.5)	1.7 (0.5)	1.6 (0.6)	1.5 (0.5)
Genital symptoms	Mean (SD)	1.6 (0.6)	1.6 (0.7)	1.5 (0.7)	1.6 (0.6)	1.6 (0.7)
Hypochondriasis	Mean (SD)	0.7 (0.6)	0.9 (0.8)	0.9 (1.0)	0.5 (0.8)	0.8 (0.8)
Loss of weight according to patient	Mean (SD)	0.4 (0.7)	0.6 (0.9)	0.5 (0.9)	0.3 (0.8)	0.5 (0.8)
Insight	Mean (SD)	0.4 (0.5)	0.4 (0.5)	0.3 (0.5)	0.5 (0.5)	0.4 (0.5)

Abbreviations: HAM‐D17, 17‐item Hamilton Rating Scale for Depression; SD, standard deviation.

### Contribution of individual HAM‐D17 items to Cluster 4

**Figure 3:**
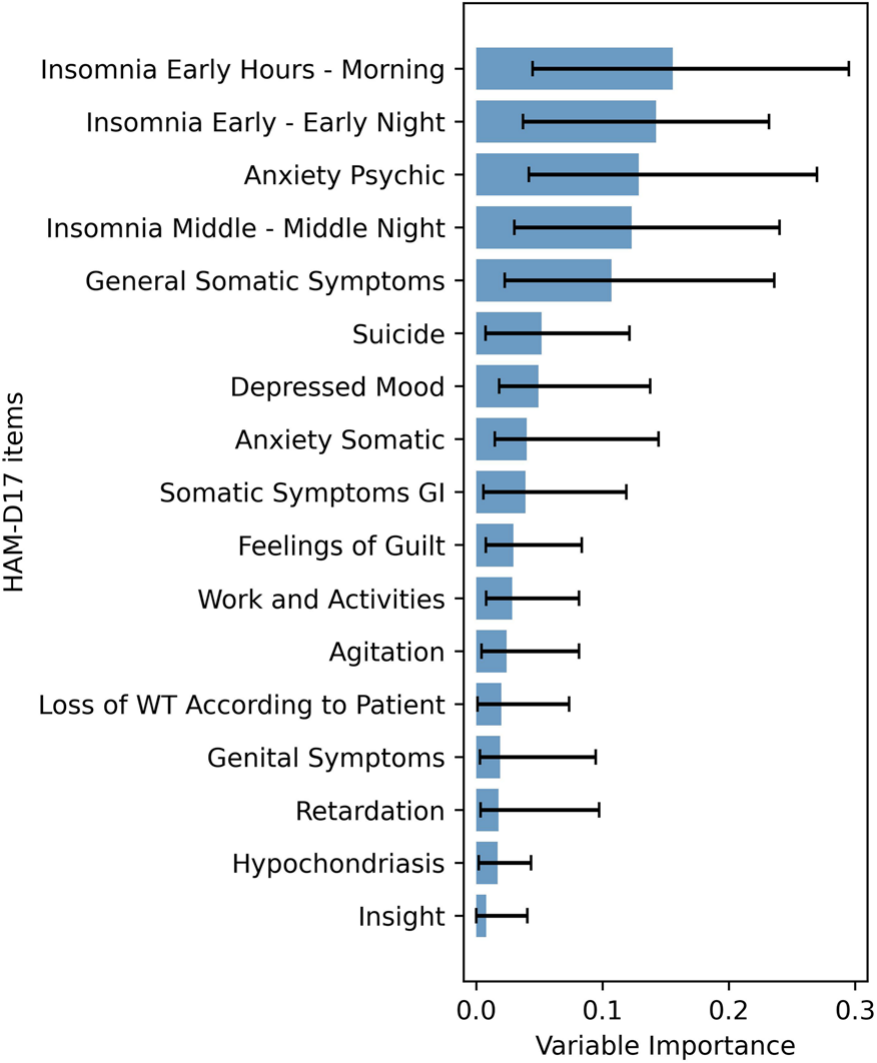
Variable importance of 17‐item Hamilton Rating Scale for Depression (HAM‐D17) items changes from Day 15 to 22 for Cluster 4 identification. Error bars indicate 95% confidence intervals for variable importance derived from 1000 bootstrap resamples. GI, gastrointestinal; WT, weight.

**Figure 4:**
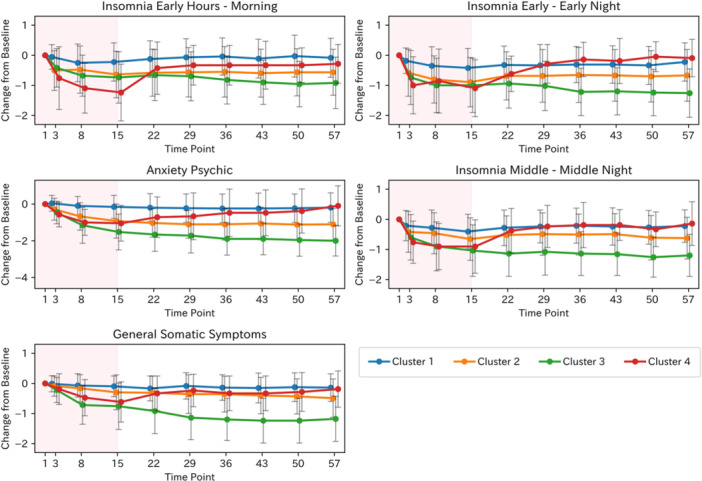
Mean trajectories of top 5 17‐item Hamilton Rating Scale for Depression (HAM‐D17) items by Cluster for Cluster 4 identification. The shaded red area indicates the treatment period (Days 1–15). Error bars represent standard deviation.

Given the distinctive symptom worsening pattern of Cluster 4, we aimed to estimate the symptom items associated with Cluster 4. Specifically, we trained a Random Forest classifier using Cluster 4 or otherwise (Clusters 1–3) as the outcome and the change from baseline of each HAM‐D17 item from Day 15 to Day 22 as predictors, since an increase in the HAM‐D17 total score was observed from Day 15 in Cluster 4. The variable importance scores are shown in Figure 3. The five items with the highest importance were insomnia early hours, insomnia early, insomnia middle, anxiety psychic, and general somatic symptoms. All three insomnia‐related symptoms were included in the top five symptoms, suggesting that worsening insomnia‐related symptoms were a major characteristic of Cluster 4. The mean trajectories of these five items are shown in Figure 4; each showed an increase in Cluster 4 after Day 15. The trajectories for the remaining items are shown in Figure S4.

### PHQ‐9 trajectories by cluster

**Figure 5:**
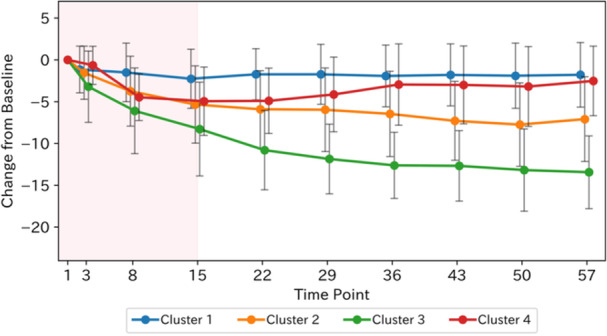
Trajectories of change from baseline in Patient Health Questionnaire‐9 (PHQ‐9) total score by cluster. The shaded red area indicates the treatment period (Days 1–15). Error bars represent standard deviation.

The mean PHQ‐9 total scores by cluster are shown in Figure 5. Cluster 4 exhibited an increase in the PHQ‐9 total score post‐Day 15, which parallels the results observed with the HAM‐D17 total scores. The trajectories for the PHQ‐9 item “Trouble Falling or Staying Asleep” increased during the follow‐up period in Cluster 4 (Figure S5), consistent with the insomnia‐related HAM‐D17 findings.

## DISCUSSION

Time‐series clustering of the change from baseline HAM‐D17 total scores in the zuranolone 30 mg group identified four distinct symptom trajectory clusters. Three clusters showed differing magnitudes of symptom reduction over the 2‐week dosing period and maintained symptom levels at the end of dosing throughout the 6‐week follow‐up phase. The fourth cluster exhibited a mean decrease in HAM‐D17 total scores during treatment but a mean increase in scores after dosing ceased. Baseline demographics, HAM‐D17 total scores, episode duration, and other clinical variables did not meaningfully differ across clusters. Feature importance analysis using a Random Forest classifier estimated five symptoms associated with Cluster 4, which showed a trend toward symptom worsening during the post‐dosing follow‐up period, and revealed that insomnia‐related HAM‐D17 items were the primary drivers of cluster separation. In Cluster 4, insomnia scores notably worsened during the first week of the follow‐up period, mirroring the overall increase in depressive symptoms. We considered predicting whether patients had a Cluster 4 trajectory based on the total change in the 5 HAM‐D17 items estimated by Random Forest from Day 15 to Day 22. The area under the ROC curve was 0.73, and the cutoff value based on the Youden index was 2. This cutoff value provided a sensitivity of 0.57, specificity of 0.86, positive predictive value of 32%, and negative predictive value of 95%. Although only 21 patients corresponded to Cluster 4, clinically, if the deterioration in these 5 items was 1 point or less, it suggested a 95% probability that the patient's trajectory would correspond to a cluster other than Cluster 4. In the clustering analysis of the placebo arm, no trajectory pattern cluster analogous to Cluster 4 in the zuranolone arm—characterized by an increase in scores during the follow‐up period—was observed. The trajectories of HAM‐D17 and PHQ‐9 scores were generally consistent across clusters, and in Cluster 4, an increase in the insomnia‐related PHQ‐9 item score was also particularly notable during the follow‐up phase.

In general, responsiveness to antidepressant treatment varies greatly among patients with MDD, and it is considered difficult to predict treatment response based on pre‐treatment symptoms or patient background.^3^, ^12^ Therefore, in real‐world clinical practice, clinicians typically assess the patient's response to antidepressants over time and decide whether to continue the current treatment or switch to another agent. However, conventional antidepressants require several weeks to manifest their therapeutic effects, and it usually takes 6–8 weeks to determine efficacy.^13^, ^14^ In the present study, we also observed substantial inter‐individual variation in response to zuranolone, and time‐series clustering analysis identified four distinct trajectory patterns. There were no meaningful differences in patient backgrounds and clinical symptoms among these patterns, suggesting that, as with existing antidepressants, it is difficult to predict the trajectory pattern that a patient will follow prior to treatment. In contrast, given that clear differences in treatment response among the trajectory patterns were evident at the end of the 2‐week treatment period, it is suggested that the efficacy of zuranolone for each patient can be assessed at that point.

During the subsequent 6‐week follow‐up period, symptom levels remained largely stable except for Cluster 4. Referencing findings on allopregnanolone, the endogenous neurosteroid and parent compound of zuranolone, studies utilizing stress‐induced animal models of depression have reported that the stress‐related reduction of hippocampal brain‐derived neurotrophic factor (BDNF) is restored by the administration of allopregnanolone.^15^ These findings raise the possibility that modulation of neurogenesis and neuroplasticity may be involved in the sustained therapeutic effects.^16^ Further research is needed to elucidate the mechanisms underlying the stability of symptoms observed during the follow‐up phase. Given that depressive symptoms did not fluctuate substantially during the follow‐up phase, it may be feasible to determine whether to continue zuranolone treatment after a drug‐free interval based on symptom levels at the end of the initial 2‐week dosing period. In contrast, a small subset of patients exhibited a tendency toward worsening of depressive symptoms during the follow‐up period. Feature importance analysis revealed that worsening of HAM‐D17 sleep items during the first week of the follow‐up period was a major factor contributing to this trajectory. Insomnia is a critical symptom of depression, and residual sleep disturbances have been reported to increase the risk of depressive relapse.^17^, ^18^ Therefore, in patients who have achieved a clinically meaningful improvement in depressive symptoms with zuranolone, it is important to closely monitor sleep status during the initial week off medication when considering continuation of zuranolone treatment after a drug‐free interval. Early detection of insomnia exacerbation may enable prompt intervention and prevention of full relapse.

This study has several limitations. First, this analysis was post hoc and not pre‐specified in the original trial protocols. Second, because the trajectory clusters were derived from a data‐driven method, there are no clear defining criteria for each cluster; consequently some cases do not completely match the characteristics of each cluster, and the applicability of these cluster characteristics to other populations including other ethnic populations, patients with more severe illness or comorbidities excluded from clinical trials, and real‐world clinical settings where assessment completion may be less rigorous remains to be tested in future studies. Future research should focus on assessing the reproducibility and generalizability of these findings in independent cohorts, conducting a more detailed analysis of the characteristics of each trajectory cluster, and examining specific interventions for patients who exhibit symptom worsening.

## CONCLUSIONS

This study highlights the diversity of symptom trajectory patterns observed in patients treated with zuranolone and provides observational insights that may support clinical considerations and future research.

## AUTHOR CONTRIBUTIONS

Masaki Kato, Nakao Iwata, and Tomoko Motomiya: Conception and design, interpretation of results, and review of the manuscript. Saki Nakano, Takamichi Baba, and Yuto Kashiwagi: Conception and design, analysis of data, interpretation of results, drafting the manuscript, and review of the manuscript.

## CONFLICT OF INTEREST STATEMENT

M.K. has received grants from AMED and Japanese Ministry of Health, Labour and Welfare; consulting fees from Shionogi & Co., Ltd., Sumitomo Pharma Co., Ltd., Otsuka Pharmaceutical Co., Ltd., Lundbeck Japan K.K., Takeda Pharmaceutical Co., Ltd., and Nippon Chemiphar Co., Ltd.; speaker honoraria from Sumitomo Pharma Co., Ltd., Otsuka Pharmaceutical Co., Ltd., Lundbeck Japan K.K., Takeda Pharmaceutical Co., Ltd., Meiji Seika Pharma Co., Ltd., Shionogi & Co., Ltd., Mitsubishi Tanabe Pharma Corporation, Viatris Inc., Eisai Co., Ltd., and Kyowa Pharmaceutical Industry Co. Ltd.; and is in the general management committee for Depression Treatment Guidelines, Japan Society of Mood Disorder and the Vice Chairman of the Guideline Development Committee Japan Society of Mood Disorders in the past 36 months. T.B., S.N., and Y.K. are full‐time employees and own stocks via the employee stock ownership society of Shionogi & Co., Ltd. T.M. is a full‐time employee of Shionogi & Co., Ltd. N.I. has received speaker honoraria from Sumitomo Pharma Co., Ltd., Otsuka Pharmaceutical Co., Ltd., and Takeda Pharmaceutical Co., Ltd.

## ETHICS APPROVAL STATEMENT

This study was conducted in compliance with ethical principles based on international guidelines, including the Declaration of Helsinki, Council for International Organizations of Medical Sciences, International Ethical Guidelines, ICH‐GCP guidelines, and other applicable laws and regulations, and was approved by the institutional review board and ethics committee of each study site.^6^

## PATIENT CONSENT STATEMENT

Written informed consent was obtained from all participants or their legally authorized representatives after a full explanation of the study procedures and objectives.

## CLINICAL TRIAL REGISTRATION

The study was registered with the Japan Registry for Clinical Trials (jRCT) Clinical Trials Registry before patient enrollment (study IDs: jRCT2080225180 and jRCT2031210577).

## Supporting information

Supporting Information.

## Data Availability

Shionogi & Co., Ltd. is committed to disclosing the synopses and results of its clinical trials and sharing clinical trial data (raw dataset or study data tabulation model dataset) with researchers upon request. If the research proposal is reviewed and approved by an independent review panel, anonymized data and redacted documents will be provided in a secure research environment. For further details, please refer to the websites of Shionogi & Co., Ltd. (https://www.shionogi.com/global/en/company/policies/shionogi‐group‐clinical‐trial‐data‐transparency‐policy.html) and Vivli (https://vivli.org/).

## References

[1] Ribeiro JD , Huang X , Fox KR , Franklin JC . Depression and hopelessness as risk factors for suicide ideation, attempts and death: meta‐analysis of longitudinal studies. Br J Psychiatry. 2018;212(5):279–286. 10.1192/bjp.2018.27 29587888

[2] Kawakami N , Tachimori H , Takeshima T . Large‐scale epidemiological survey study on the prevalence of mental disorders: World Mental Health Japan 2nd Survey. Available from: https://mhlw‐grants.niph.go.jp/project/24202 (in Japanese).

[3] Sakurai H , Uchida H , Kato M , Suzuki T , Baba H , Watanabe K , et al. Pharmacological management of depression: Japanese expert consensus. J Affect Disord. 2020;266:626–632. 10.1016/j.jad.2020.01.149 32056937

[4] Zuranolone prescribing information . Available from: https://www.accessdata.fda.gov/drugsatfda_docs/label/2023/217369s000lbl.pdf

[5] Summary of product characteristics. Zurzuvae product information. Available from: https://www.ema.europa.eu/en/documents/product‐information/zurzuvae‐epar‐product‐information_en.pdf. Annex I.

[6] Kato M , Nakagome K , Baba T , Sonoyama T , Okutsu D , Yamanaka H , et al. Efficacy and safety of zuranolone in Japanese adults with major depressive disorder: a double‐blind, randomized, placebo‐controlled, Phase 2 clinical trial. Psychiatry Clin Neurosci. 2023;77(9):497–509. 37252829 10.1111/pcn.13569 PMC11488630

[7] Kato M , Nakagome K , Baba T , Sonoyama T , Fukuju H , Shimizu R , et al. Efficacy and safety of zuranolone in Japanese adults with major depressive disorder: a double‐blind, randomized, placebo‐controlled, Phase 3 clinical trial. Psychiatry Clin Neurosci. 2026;80(1):76–86. 10.1111/pcn.13917 41251319 PMC12757765

[8] Shionogi receives approval in Japan to manufacture and market ZURZUVAE® capsules 30 mg for the treatment of major depressive disorder. Available from: https://www.shionogi.com/globaltml/en/news/2025/12/E_20251222.html

[9] Tavenard R , Faouzi J , Vandewiele G . Tslearn, a machine learning toolkit for time series data. J Mach Learn Res. 2020;21(118):1–6.34305477

[10] Mehdipour Ghazi M , Urdanibia‐Centelles O , Bakhtiari A , Fagerlund B , Vestergaard MB , Larsson H , et al. Cognitive aging and reserve factors in the Metropolit 1953 Danish male cohort. GeroScience. 2025;47(2):2475–2493. 39570569 10.1007/s11357-024-01427-2 PMC11978600

[11] Yao L , Li Q , Zhou Z , Yin J , Wang T , Liu Y , et al. Machine learning models for predicting multimorbidity trajectories in middle‐aged and elderly adults. Sci Rep. 2025;15(1):24711. 40634428 10.1038/s41598-025-07060-z PMC12241554

[12] Trivedi MH , Rush AJ , Wisniewski SR , Nierenberg AA , Warden D , Ritz L , et al. Evaluation of outcomes with citalopram for depression using measurement‐based care in STAR*D: implications for clinical practice. Am J Psychiatry. 2006;163(1):28–40. 16390886 10.1176/appi.ajp.163.1.28

[13] Rush AJ , Trivedi MH , Wisniewski SR , Nierenberg AA , Stewart JW , Warden D , et al. Acute and longer‐term outcomes in depressed outpatients requiring one or several treatment steps: a STAR*D report. Am J Psychiatry. 2006;163(11):1905–1917. 17074942 10.1176/ajp.2006.163.11.1905

[14] Gaynes BN , Warden D , Trivedi MH , Wisniewski SR , Fava M , Rush AJ . What did STAR*D teach us? Results from a large‐scale, practical, clinical trial for patients with depression. Psychiatr Serv. 2009;60(11):1439–1445. 19880458 10.1176/ps.2009.60.11.1439

[15] Evans J , Sun Y , Mcgregor A , Connor B . Allopregnanolone regulates neurogenesis and depressive/anxiety‐like behaviour in a social isolation rodent model of chronic stress. Neuropharmacology. 2012;63(8):1315–1326. 22939998 10.1016/j.neuropharm.2012.08.012

[16] Almeida FB , Nin MS , Barros HMT . The role of allopregnanolone in depressive‐like behaviors: focus on neurotrophic proteins. Neurobiol Stress. 2020;12(1):100218. 32435667 10.1016/j.ynstr.2020.100218 PMC7231971

[17] Inada K , Enomoto M , Yamato K , Marumoto T , Takeshima M , Mishima K . Effect of residual insomnia and use of hypnotics on relapse of depression: a retrospective cohort study using a health insurance claims database. J Affect Disord. 2021;281:539–546. 33401142 10.1016/j.jad.2020.12.040

[18] Israel JA . The impact of residual symptoms in major depression. Pharmaceuticals. 2010;3(8):2426–2440. 27713362 10.3390/ph3082426 PMC4033933

