# Supplementary material for: Analysis of depressive symptom trajectory patterns with zuranolone: Time‐series clustering of Japanese Phase 2 and Phase 3 trial data
Source: PCN Rep. 2026 Mar 13;5(1):e70320. doi: 10.1002/pcn5.70320 (PMC13097687; doi:10.1002/pcn5.70320)
Supplement: Supplementary file 1 — Supporting Information. [file PCN5-5-e70320-s001.docx]

**Supplementary data**

**Table S1** Baseline Demographic and Clinical Characteristics by Primary Analysis Set and the Patients Excluded from the Primary Analysis Set

|  |  | Primary analysis set N = 209 n (%) | Patients excluded from the primary analysis set N = 75 n (%) |
| --- | --- | --- | --- |
| Sex | Male | 99 (47.4) | 37 (49.3) |
|  | Female | 110 (52.6) | 38 (50.7) |
| Age | Mean (SD) | 39.1 (11.7) | 40.4 (14.8) |
|  | ≥ 18 to < 25 | 27 (12.9) | 15 (20.0) |
|  | ≥ 25 to < 45 | 115 (55.0) | 27 (36.0) |
|  | ≥ 45 to < 65 | 66 (31.6) | 28 (37.3) |
|  | ≥ 65 | 1 (0.5) | 5 (6.7) |
| BMI | Mean (SD) | 23.12 (4.80) | 22.02 (3.70) |
| Classification based on DSM-5 | Single Episode | 85 (40.7) | 35 (46.7) |
|  | Recurrent | 124 (59.3) | 40 (53.3) |
| Episode recurrences | First Time | 86 (41.1) | 35 (46.7) |
|  | 2nd Time | 66 (31.6) | 24 (32.0) |
|  | 3rd-7th time | 53 (25.4) | 16 (21.3) |
|  | No Less than 8 times | 2 (1.0) | 0 |
|  | Unknown | 2 (1.0) | 0 |
| Duration of current episode at randomization (month) | Mean (SD) | 6.2 (3.0) | 5.7 (2.7) |
|  | 2-4 months | 67 (32.1) | 25 (33.3) |
|  | 4-6 months | 50 (23.9) | 19 (25.3) |
|  | 6-8 months | 35 (16.7) | 15 (20.0) |
|  | 8-10 months | 23 (11.0) | 9 (12.0) |
|  | 10-12 months | 27 (12.9) | 4 (5.3) |
|  | 12 months or more | 7 (3.3) | 3 (4.0) |
| Presence or absence of prior drug for depressive episodes | Yes | 114 (54.5) | 44 (58.7) |
|  | No | 95 (45.5) | 31 (41.3) |

Duration of the current episode at randomization = (Date of randomization) – (Onset date) + 1.

Abbreviations: BMI, body mass index; HAM-D17, 17-item Hamilton Rating Scale for Depression; PHQ-9, Patient Health Questionnaire-9; DSM-5, Diagnostic and Statistical Manual of Mental Disorders, fifth edition; SD, standard deviation.

**Table S2** Baseline Clinical Characteristics by Primary Analysis Set and Patients Excluded from the Primary Analysis Set

| Baseline values of HAM-D17 | |  | Primary analysis set N = 209 n (%) | | | Patients excluded from the primary analysis set N = 75 n (%) |  |  |
| --- | --- | --- | --- | --- | --- | --- | --- | --- |
| Total Score | Mean (SD) | | | 24.2 (2.0) | 24.5 (2.2) | | |  |
| Depressed Mood | Mean (SD) | | | 2.9 (0.6) | 3.1 (0.7) | | |  |
| Feelings of Guilt | | Mean (SD) | | | 1.6 (0.8) | 1.6 (0.7) | | |
| Suicide | | Mean (SD) | | | 0.7 (0.7) | 0.8 (0.7) | | |
| Insomnia Early - Early Night | | Mean (SD) | | | 1.6 (0.6) | 1.4 (0.7) | | |
| Insomnia Middle - Middle Night | | Mean (SD) | | | 1.4 (0.6) | 1.4 (0.6) | | |
| Insomnia Early Hours - Morning | | Mean (SD) | | | 1.3 (0.7) | 1.4 (0.6) | | |
| Work and Activities | | Mean (SD) | | | 2.9 (0.7) | 2.8 (0.7) | | |
| Retardation | | Mean (SD) | | | 1.2 (0.7) | 1.2 (0.7) | | |
| Agitation | | Mean (SD) | | | 0.9 (0.7) | 0.8 (0.7) | | |
| Anxiety Psychic | | Mean (SD) | | | 2.4 (0.8) | 2.6 (0.8) | | |
| Anxiety Somatic | | Mean (SD) | | | 1.7 (0.7) | 1.7 (0.6) | | |
| Somatic Symptoms Gastrointestinal | | Mean (SD) | | | 0.9 (0.6) | 0.9 (0.6) | | |
| General Somatic Symptoms | | Mean (SD) | | | 1.5 (0.5) | 1.7 (0.5) | | |
| Genital Symptoms | | Mean (SD) | | | 1.6 (0.7) | 1.5 (0.7) | | |
| Hypochondriasis | | Mean (SD) | | | 0.8 (0.8) | 0.9 (0.9) | | |
| Loss of Weight According to Patient | | Mean (SD) | | | 0.5 (0.8) | 0.3 (0.7) | | |
| Insight | | Mean (SD) | | | 0.4 (0.5) | 0.4 (0.5) | | |

HAM-D17, 17-item Hamilton Rating Scale for Depression; SD, standard deviation.


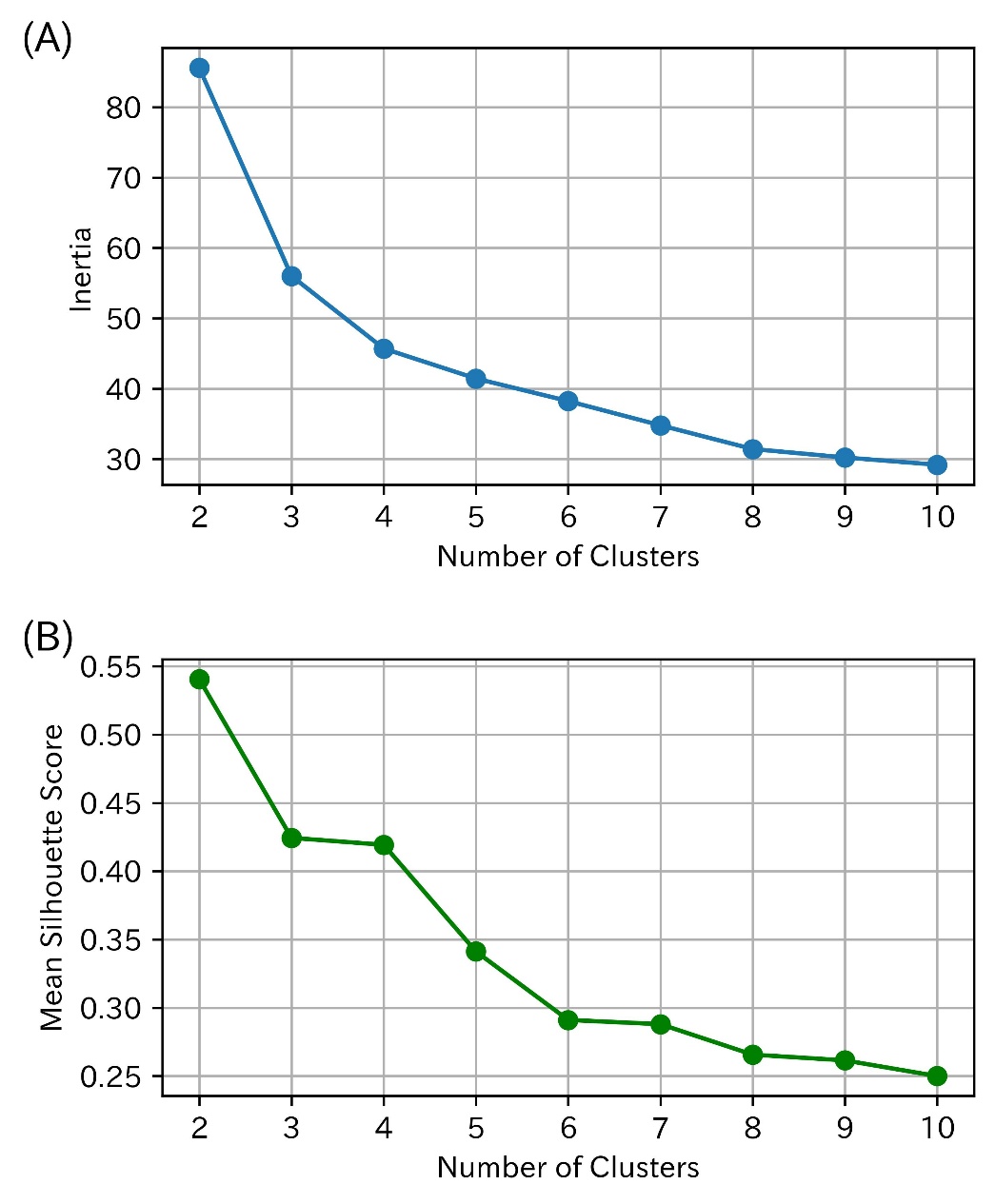


**Figure S1** Determining Optimal Cluster Number in the Zuranolone 30 mg Arm

(A) Elbow Method. (B) Silhouette Method.

Within-cluster sum of squares (inertia) and mean silhouette scores plotted against the number of clusters (k).


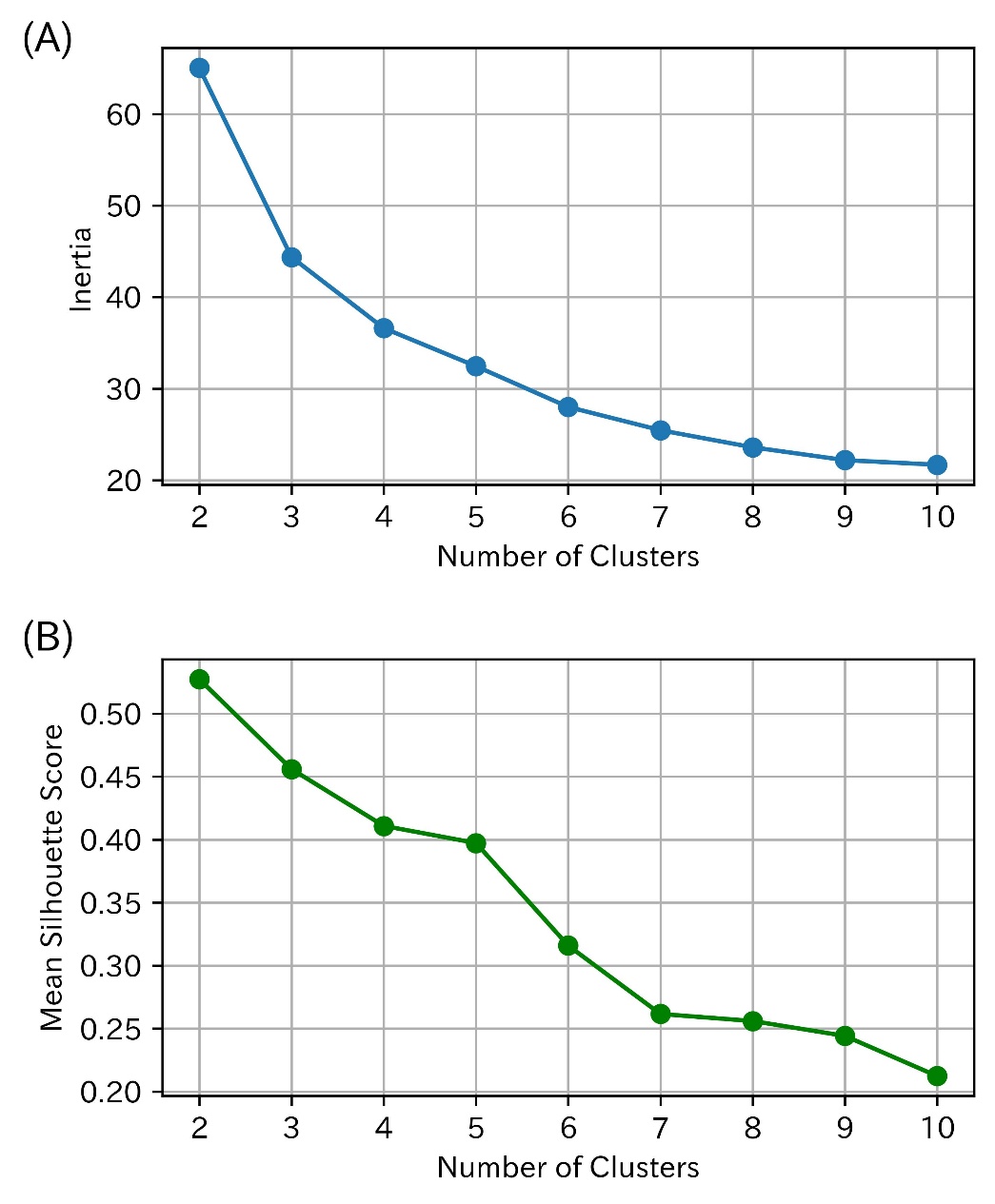


**Figure S2** Determining Optimal Cluster Number in the Placebo Arm

(A) Elbow Method. (B) Silhouette Method.

Within-cluster sum of squares (inertia) and mean silhouette scores plotted against the number of clusters (k).


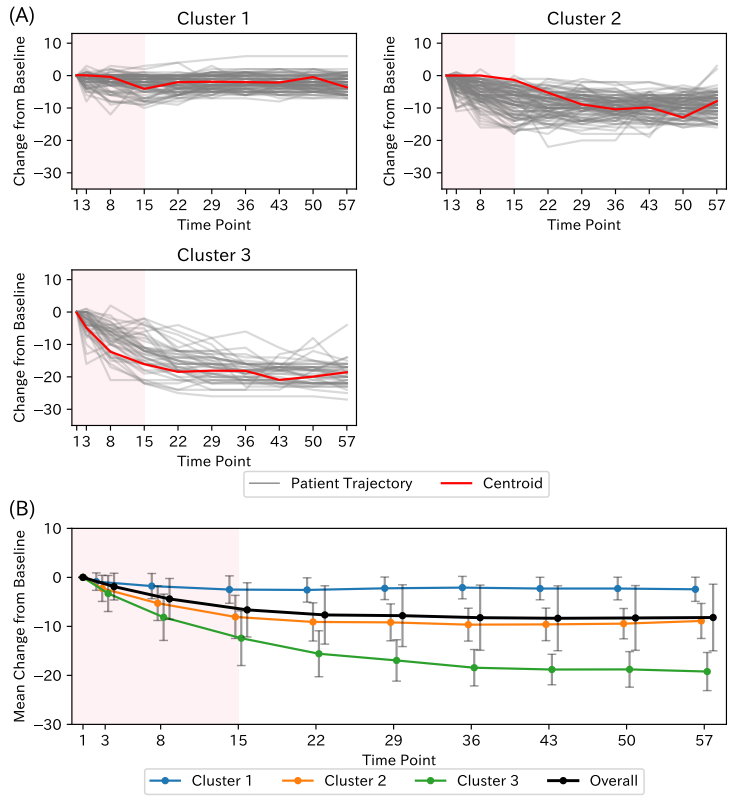


**Figure S3** Clustering of 17-item Hamilton Rating Scale for Depression (HAM-D17) Change from Baseline Trajectories in the Placebo Arm

(A) Individual trajectories and cluster centroids. (B) Mean trajectory for each cluster.

Cluster 1 (n=90), Cluster 2 (n=94), Cluster 3 (n=41). The shaded red area indicates the treatment period (Days 1–15). Error bars in panel B represent standard deviation.


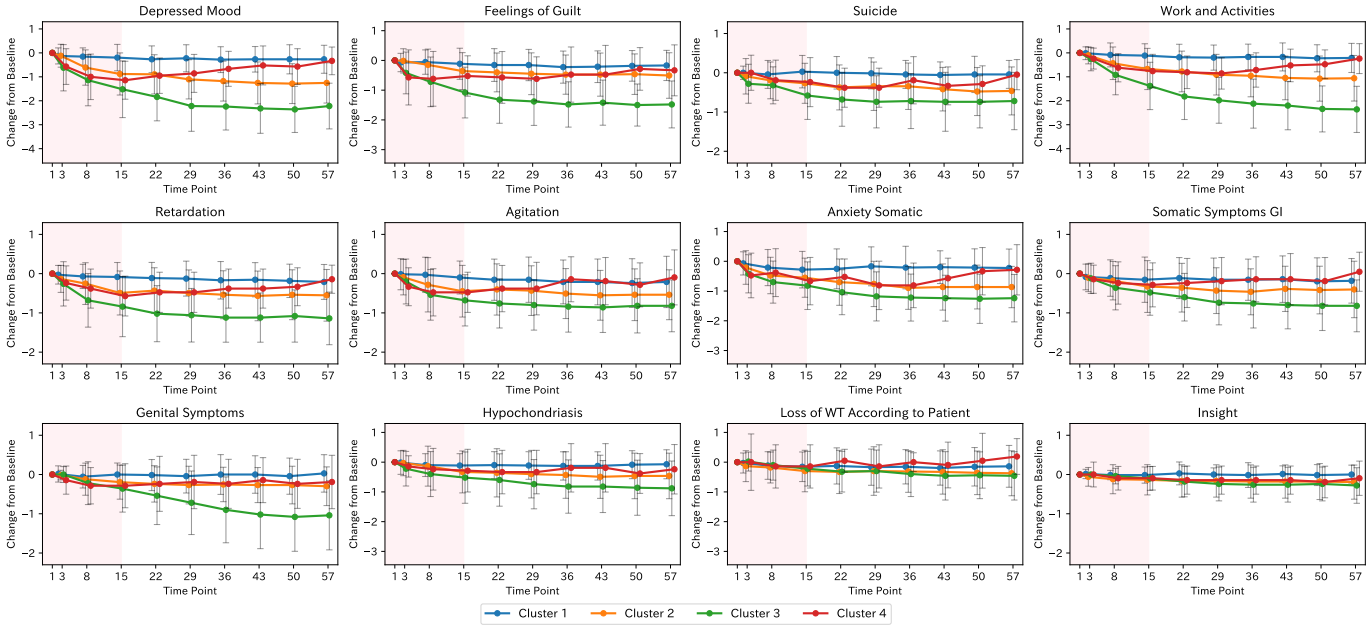


**Figure S4** Mean Trajectories of Other 17-item Hamilton Rating Scale for Depression (HAM-D17) Items by Cluster

Mean trajectories of HAM-D17 items not included in Figure 4. The shaded red area indicates the treatment period (Days 1–15). Error bars represent standard deviation.

**
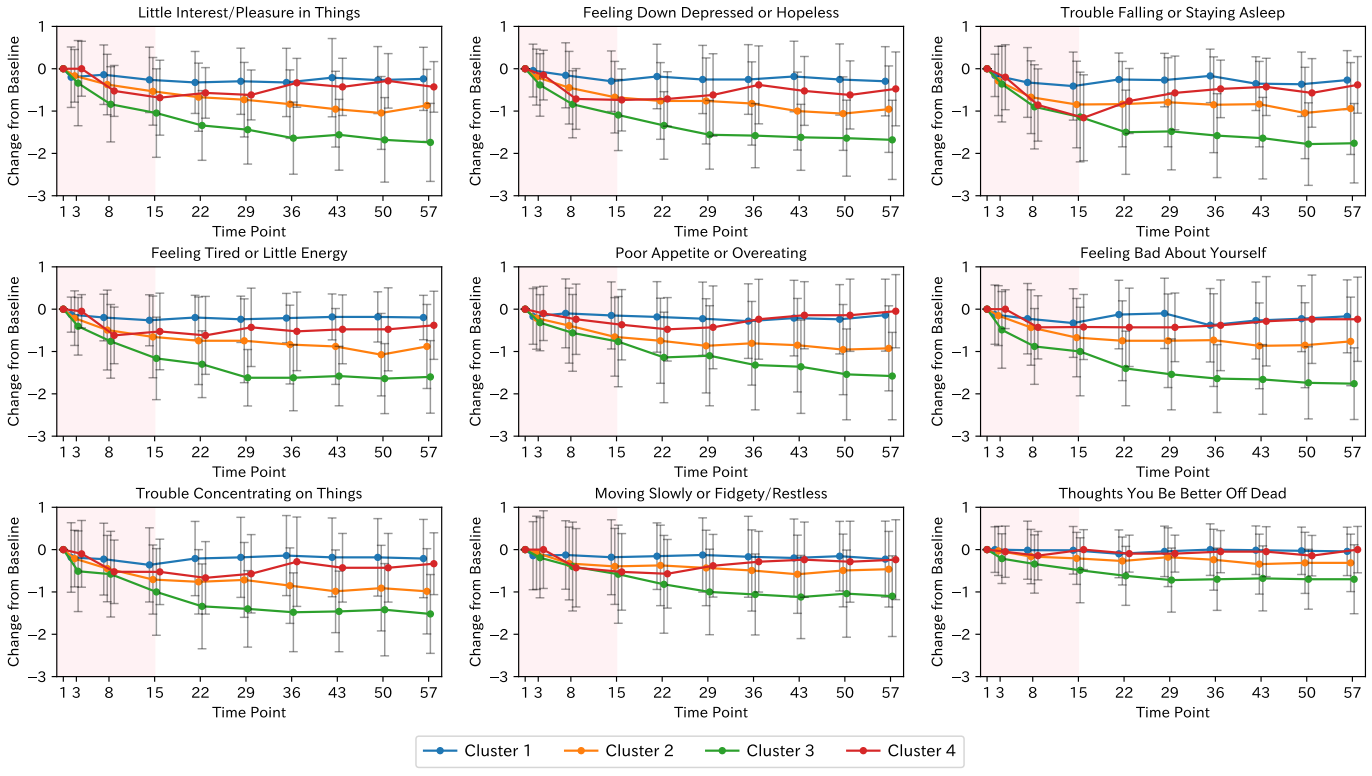
**

**Figure S5** Trajectories of Change from Baseline in Patient Health Questionnaire-9 (PHQ-9) Items by Cluster

The shaded red area indicates the treatment period (Days 1–15). Error bars represent standard deviation.
